# Self-assembled chitosan nanoparticles for intranasal delivery of recombinant protein interleukin-17 receptor C (IL-17RC): preparation and evaluation in asthma mice

**DOI:** 10.1080/21655979.2021.1940622

**Published:** 2021-06-28

**Authors:** Yongli Lv, Jianhua Zhang, Chaoying Wang

**Affiliations:** a Department of Paediatrics, Xinhua Hospital Affiliated To Shanghai Jiao Tong University School Of Medicine, Shanghai, China; b Department of Paediatrics, Shanghai Jiao Tong University Affiliated Sixth People’s Hospital, Shanghai, China

**Keywords:** Chitosan nanoparticles, intranasal delivery, recombinant protein, IL-17RC, asthma

## Abstract

Asthma is mentioned as a chronic airway inflammatory disease, whose pathogenesis is complicated. The promotion of inflammation in asthma by IL-17A and IL-17F has been confirmed. In addition to covalent homodimers, both cytokines are also able to form heterodimers, further inducing downstream pathways via binding to the IL-17RA and IL-17RC receptor complex. In recent years, IL-17RA and its signal transduction pathway have been extensively researched. IL-17RC, however, remains relatively unexplored. In the present study, we self-assembled chitosan (CS) nanoparticles for intranasal delivery of recombinant protein IL-17RC (rIL-17RC) and preliminarily investigated its effect on a murine model of allergic asthma induced by ovalbumin (OVA). rIL-17RC was produced by the prokaryotic expression system and encapsulated into the CS nanoparticles via ionic cross-linking technique. The results showed that CS-RC nanoparticles via intranasal intervention significantly caused inhibition of mucus secretion and airway inflammatory cell infiltration, and reduced IL-4, IL-17, IL-17F levels in BALF. Hence, delivering receptor proteins such as IL-17RC, through CS nanoparticles as a carrier, could be an attractive therapeutic intervention for asthma.

## Grafical abstract

**Figure:**
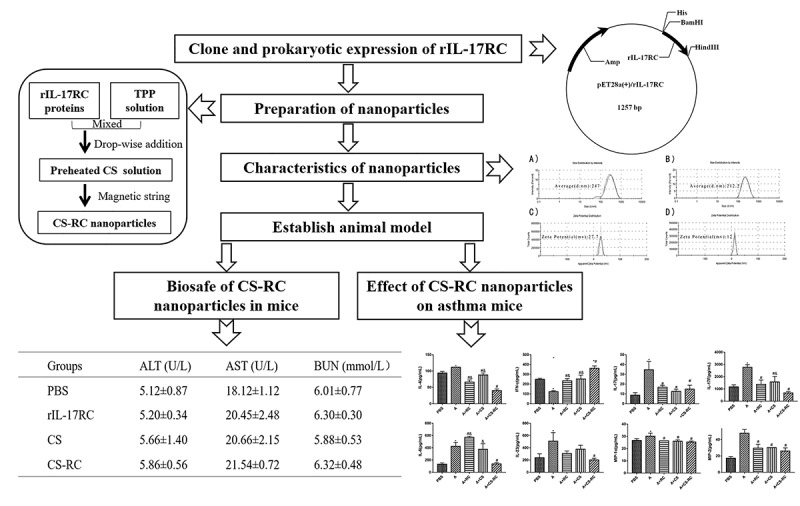

## Highlights

1. Chitosan-rIL-17RC (CS-RC) nanoparticles inhibit mucus secretion via intranasal intervention in asthma.
2. CS-RC nanoparticles inhibit airway inflammatory cell infiltration.
3. CS-RC nanoparticles reduced IL-4, IL-17, IL-17F levels in BALF of asthma.

## 1. Introduction

As being mentioned as a chronic airway inflammatory disease, asthma shares major characteristics of airway hyperresponsiveness, mucus overproduction, airway wall remodeling [1]. It has a profound affection on human health with a prevalence of 7–10% worldwide, among which approximately 70–80% patients suffering from allergic asthma [2]. T-helper 2 (Th2) cells are the main mediators of allergic asthma. However, with the newly discovered third subset of T-helper cells, namely Th17 cells, the Th1-Th2 paradigm is updated [3–5]. In mouse models of asthma, the involvement of Th17 cells in neutrophilic airway inflammation and Th2 cell-mediated eosinophilic airway inflammation has been confirmed [6]. The main cytokines of Th17 cells produce are interleukin (IL)-17A (or IL-17) and IL-17F. Both of the two cytokines are involved in the pathogenesis of allergic asthma [2,4,7–9], whose expression is increased in bronchial specimens from allergic asthma patients [10]. They can form either covalent homodimers or IL-17A/IL-17F heterodimers, then trigger downstream pathways via binding to the same receptor complex comprising IL-17RA and IL-17RC. Hence, targeting IL-17 receptor could be a potential therapeutic intervention for asthma.

Among the IL-17 receptor subfamily (IL-17RA-IL-17RE), IL-17RA is shared by at least four ligands as receptor subunit, while IL-17RC is specific for IL-17A and IL-17F [11]. Given IL-17RA has been researched, we focused on IL-17RC protein. IL-17RC protein is a single-pass type I transmembrane protein, comprised of intracellular similar expression to fibroblast growth factor genes (SEF)/IL-17R (SEFIR) domain, extracellular-fibronectin III-like (FnIII) domain, and transmembrane domain [12]. It shares only 22% amino acid sequence homology with IL-17RA, which leads to a considerable difference in their tissue distribution [13–15]. For example, IL-17RC expresses high level in the epithelium of bronchus and lung; however, a low level of IL-17RA is detected in the lung [13,16]. Considering that previous report has implied the existence of soluble IL-17R proteins lacking transmembrane and intracellular domains [15], we try to use a prokaryotic expression system to produce and purify soluble mouse IL-17RC (mIL-17RC) extracellular domain, then valuate its effect *in vivo*. In this study, recombinant plasmid pET28a (+)/mIL-17RC was constructed via ligation-independent cloning. After identification, the verified pET28a (+)/mIL-17RC plasmid was transferred into expression host *Escherichia coli* (*E. coli*). The rIL-17RC protein was expressed in this prokaryotic expression system and then purified via nickel-affinity chromatography, urea gradient dialysis, ultrafiltration concentration and endotoxin removal.

However, applying proteins *in vivo* alone via traditional administration routes may not achieve the ideal effect, due to their low oral bioavailability or low compliance after repeatedly invasive injections [17]. Hence, extensive researches have been conducted to explore alternative routes. Intranasal administration of protein drugs is attractive and convenient, not only because it avoids first-pass metabolism in the liver, but also because in this way, the absorptive surface area is relatively large and proteolytic enzyme activity is low [18–20]. Nevertheless, appropriate carriers are needed to prevent the rapid clearance by nasal mucociliary and overcome the limitation of low permeability through the mucosal barriers, thus enhancing the absorption of proteins through the mucosal barriers [21].

Among the carriers applied to the mucosal delivery system, chitosan (CS) has attracted more and more attention in recent years. In addition to the advantages of biocompatibility, biodegradability, non-toxicity, and ease of preparation, it also owns mucoadhesive and absorption-enhancing properties, which makes it an ideal candidate for the delivery of proteins [22].

To date, CS nanoparticles as IL-17RC protein vehicles for nasal administration of asthma mice have not yet been reported. We hypothesized that soluble IL-17RC protein delivered by CS nanoparticles would have the positive effect on asthma, alleviating airway inflammation induced by IL-17 and IL-17F signal pathway. Thus, in our study, we aimed to extract pure soluble rIL-17RC proteins via prokaryotic expression system, then encapsulated these proteins into the chitosan nanoparticles via ionic cross-linking technique and ultimately investigated the effect of CS-RC nanoparticles on asthma mice.

## 2. Materials and methods

### 2.1 Bacterial strains, chemicals, and animals

*E. coli* BL21 (DE3) and *E. coli* DH5α were purchased from Tiangen biotech (Beijing, China) and Sangon (Shanghai, China), respectively. pET28a vector was a kind gift from Shanghai Public Health Clinical Center.

CS (Low molecular weight, deacetylation degree 75–85%), sodium tripolyphosphate (TPP) and ovalbumin (OVA) were purchased from Sigma (USA). Ultrapure water (PALL Cascada I, USA) was used throughout this experiment. All other chemicals utilized in this study were of analytical grade.

Female BALB/c mice (age: 6–8 weeks) acquired from B&K Universal Group Ltd. (Shanghai, China) were provided an OVA-free diet and kept in a specified pathogen-free (SPF) area. This study was carried out based on the approval by the Ethics Committee of Shanghai Public Health Clinical Center (Permit Number: 2018–0006).

### 2.2 Expression and purification of recombinant protein IL-17RC

According to mice IL-17RC gene sequence (NM_134159. 4), specific forward (F) and reverse (R) primer were designed, F: 5'-CTAGGATCCGAGAGACTGATGGAGCCT-3', R: 5'-AGTAAGCTTGCGCCTGTGGATGTACTTGT-3', containing BamHI and HindIII restriction enzyme sites, respectively. Using mice genomic cDNA (synthesized by our laboratory) as the template, IL-17RC coding region sequence without its signal sequence was amplified by polymerase-chain reaction (PCR) with a length of 1257-bp. Then IL-17RC gene fragment linked with pUCm-T vector via T4 DNA ligase, further was cloned into pET28a *E. coli* TOP10 strains. After blue-white screening, transformants with correct target gene fragment underwent endonuclease digestion. The next step was to separate these products by agarose gel electrophoresis and purified according to DNA gel extraction kit (Bio Teke, Beijing, China). To generate recombinant plasmid pET28a (+)/mIL-17RC, IL-17RC gene fragment linked with pET28a vector via T4 DNA ligase. On completion of transfection of recombinant plasmid into *E. coli* DH5α, colony PCR, endonuclease digestion, and gene sequencing were utilized for confirmation. The verified pET28a (+)/mIL-17RC plasmid was cloned into expression host *E. coli* BL21(DE3).

By utilizing isopropyl β-D-thiogalactoside (IPTG) at a final concentration of 1 mM, rIL-17RC expression was induced and subsequently analyzed by 10% SDS-PAGE. The purification of rIL-17RC protein used a Gravity-flow Column prepacked with an appropriate amount of Ni-NTA resin (Sangon, Shanghai, China), then eluted His-tagged proteins from the resin with two resin-bed volumes of Elution Buffer and dialyzed at 4 °C by decreasing urea gradient ranged from 6 M, 5 M, 4 M, 3 M, 2 M, 1 M to 0 M in PBS for 4 h respectively. After the dialyzed protein underwent ultrafiltration concentration, endotoxin removal, rIL-17RC was quantified by BCA protein assay (Thermo Scientific, USA) and stored in aliquots at −20°C for further analysis.

### 2.3 Western blot identification of rIL-17RC protein

Western blotting was applied to conform the His-tag fused to rIL-17RC proteins. Initially, on completion of separation using 10% SDS-PAGE, the purified proteins were blotted onto polyvinylidene fluoride (PVDF) membranes (Millipore), and subsequently 5% nonfat dry milk for blocking step. After that, overnight incubation in anti-6 × His Tag mouse monoclonal antibody (Sangon, Shanghai, China) at a dilution of 1:5000 at 4°C was performed. Following repeated washes with TBS-Tween buffer for 4 times, HRP-conjugated goat antimouse IgG (1:8000; Sangon, Shanghai, China) were used as secondary antibody. After thorough washing, the membrane was detected with a highly sensitive ECL luminescence reagent (Sangon, Shanghai, China) according manufacturer’s instructions.

### 2.4 Binding assay of rIL-17RC protein

The ELISA system was used to evaluate binding assay of rIL-17RC protein *in vitro*. In brief, the microtiter plate was coated with recombinant murine IL-17A (PeproTech, USA), followed by overnight incubation at 4°C. After being rinsed twice utilizing PBST (PBS with 0.05% Tween-20), the plate was blocked with 0.25% bovine serum albumin (BSA) in PBST. Biotin labeling reaction of rIL-17RC protein was prepared based on the instructions of Sulfo-NHS-LC-Biotin (Thermo Fisher, USA). After washing the plate three times and addition of biotinylated rIL-17RC protein to the plate, 2 h incubation was carried out at room temperature. Recombinant murine IL-17A was replaced with coated solution and rIL-17RC protein was replaced with sample diluent in the control wells. The desired color developed after adding of HRP-conjugated streptavidin and substate solution. The stop solution was added after sufficient color development. The final step was to utilize a plate reader to measure the absorbance (450 nm).

### 2.5 Preparation of nanoparticles

Preparation of CS nanoparticles by ionic cross-linking technique was completed as previous reports with slight modifications [23–25]. Briefly, by dissolving CS in acetic aqueous solution (1%, v/v), CS solution (0.2%, w/v) was obtained and then adjusted its PH to 5.0 and passed through 0.22 μm syringe filter (Millipore, USA). By dissolving sodium tripolyphosphate in ultrapure water, TPP solution (0.1%, w/v) was obtained and then also passed through 0.22 μm syringe filter. A water bath at 60°C for 10 min contributed to preheat the CS solution; ultrapure water was added into 1.2 ml TPP solution until the final mix volume to 2 ml. Subsequently, the opalescent CS nanosuspension was formed spontaneously by adding 2 ml mixed TPP solution dropwise into 3 ml preheated CS solution, under magnetic stirring (1000 rpm/min) at 4°C for 2 h. To prepare CS-RC nanoparticles, 0.4 ml rIL-17RC (1 mg/mL) was mixed with 1.2 mL TPP solution, and with the addition of ultrapure water, the mixture was adjusted to 2 mL; the rest procedures were performed the same as CS nanoparticles. On completion of centrifugation at 15,000 rpm for 30 min at 4°C, the CS and CS-RC nanoparticles were collected and subsequently resuspended in ultrapure water for their nasal mucosal intervention.

### 2.6 Characterization of nanoparticles

The average size, surface charge and polydispersity index were measured at 25°C by Zeta sizer Nano series (Malvern, UK). The observation of the morphology of nanoparticles was completed utilizing transmission electron microscopy (TEM).

To determine the encapsulation efficiency (EE) of rIL-17RC-loaded CS nanoparticles, 1 ml of CS-RC and CS nanosuspension were centrifuged at 15,000 g for 30 min. After that, Micro BCA Protein Assay Kit (Thermo Fisher, USA) allowed the determination of rIL-17RC protein amount in the collected supernatant. Finally, the formula for calculation of EE was as follows [26]: EE (%) = [(total amount of rIL-17RC – rIL-17RC amount in supernatant)/total amount of rIL-17RC]×100.

### 2.7 Experimental protocol and sample collection

To assess whether CS-RC nanoparticles accumulated *in vivo*, BALB/C mice were assigned at random into 4 groups (n = 6 per group) with different intranasal administration (every other day): 20 μl PBS, rIL-17RC proteins, CS nanoparticles, CS-RC nanoparticles. After three times intervention, we monitored their change in body weight every week. During this period, we collect the peripheral blood of mice in each group, then detected the levels of alanine aminotransferase (ALT), aspartate aminotransferase (AST) and blood urea nitrogen (BUN) in serum according to instructions of these assay kits (Jiancheng bioengineering institute, Nanjing, China). After 1 month of observation, mice were sacrificed and collected their liver, kidney, spleen, and lung for histopathological staining.

To preliminarily investigate the effect of CS-RC nanoparticles on the murine model of asthma, BALB/C mice were assigned at random into five groups (n = 6 per group): phosphate-buffered saline group (PBS), asthma group (A), asthma plus CS nanosuspension group (A+ CS), asthma plus CS-RC nanosuspension group (A+ CS-RC), asthma plus rIL-17RC group (A+ RC). The asthma model was constructed according to the previous reports [27,28], and the intervention with nanosuspension was performed as previously described with slight modifications [29]. Briefly, on day 0 and 14, all of the experimental mice, except for PBS group, were intraperitoneally injected twice with 10ug OVA and 1.6 mg aluminum hydroxide in 200ul PBS; they were then challenged with 10 mg/mL OVA via aerosol inhalation for 30 min between day 21 and 28. Intranasal intervention was given 1 day before and 1 and 3 days after the last challenge. PBS group were received PBS only. On day 32, mice were sacrificed.

Bronchoalveolar fluids (BALF) and serum were collected as previously described methods [25]. Liver, kidney, spleen, and lung tissues were isolated carefully under aseptic conditions and then fixed in 4% paraformaldehyde for histopathological examination.

### 2.8 Cytokines detection

ELISA was used to measure the related cytokines and chemokines in BALF based on the instructions. Except for MIP-1α and MIP-2 ELISA kits (Abcam), the rest of ELISA kits were purchased from eBioscience. Brief, adding the standard dilutions, samples to the corresponding wells, sample diluent was set as the blank wells. Adding prepared biotin-conjugate to all wells, the plate was incubation at room temperature for 2 hours. After washing the plate 3 times, the diluted streptavidin-HRP was then added to all wells and incubation incubated at room temperature for 1 hour. After washing the plate 3 times, the TMB substrate solution was added to all wells and incubated at room temperature. When the highest standard had developed a dark blue color, the stop solution was added into each well. The results were read at absorbance of 450 nm. The concentrations of samples were calculated from standard curve drawn by the mean absorbance of each standard concentration.

### 2.9 Histopathological analysis

On preparation of the lung tissues sections stained with Hematoxylin and eosin (H&E) or periodic acid Schiff (PAS), the sections were photographed and scored according to previously described [28,30]. In brief, the quantitative score for peribronchial inflammatory infiltration ranged from 0 to 4. A score of 0 represented none of inflammatory cell infiltration around bronchus. A score of 1 indicated occasionally, cufflike inflammatory cell infiltration. A score of 2 implied that inflammatory cell infiltration could be seen around most of the bronchi, which were surrounded by monolayer cells. A score of 3 was assigned when most bronchi were surrounded by inflammatory cells that were ringed with two to four cell layers of cells. When the number of surrounding layers was more than four, it was scored as 4. The integrate optical density (IOD) in the positive area of PAS stain was counted and then corrected by the perimeter of the corresponding airway basement membrane for standardization. Three sections were observed for each animal, and six visual fields were randomly selected for each section. Assessment of the results was completed utilizing Image-Pro Plus 6.0 (Media Cybernetics, Bethesda, MA, USA).

### 2.10 Statistical analysis

GraphPad Prism program 7.0 (San Diego, CA, USA) was adopted for statistical analysis. One-way ANOVA or Student’s t-test was utilized for comparison between groups, followed by Tukey's t-test. Mean ± standard deviation (SD) was the final form to present the results. A statistically significant difference could be suggested if P < 0.05.

## 3. Results

### 3.1 Expression and identification of rIL-17RC

**Figure 1.**
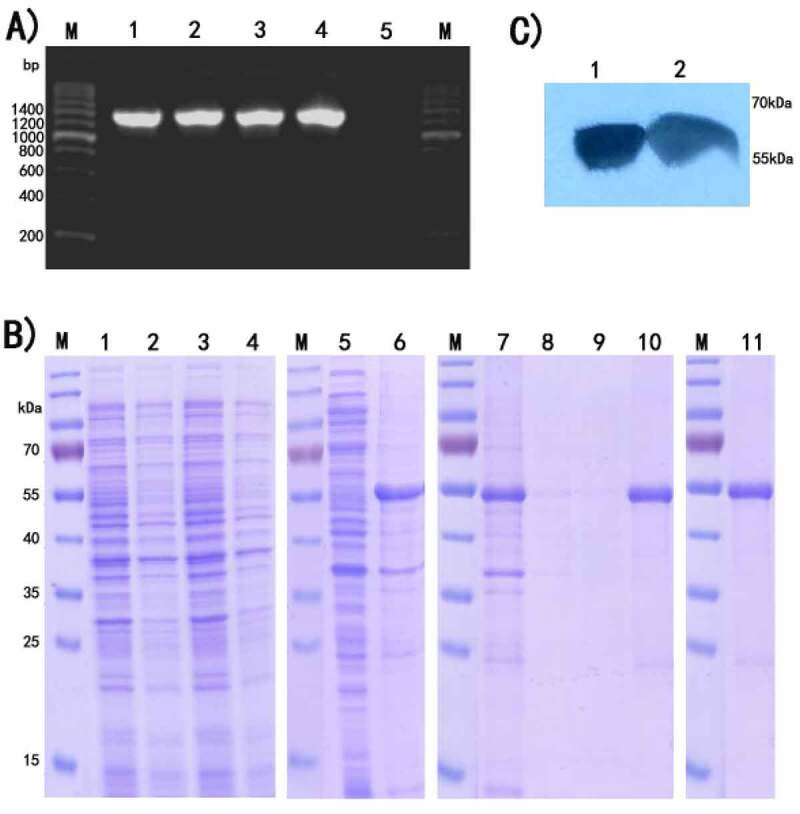
Expression and identification of rIL-17RC protein

The target gene via pET28a vector was cloned into *E. coli* BL21(DE3), followed by identification by colony PCR and agarose gel electrophoresis. As shown in Figure 1a, a uniform band could be seen at the expected molecular weight of 1300 bp. After transformant induced by IPTG and analyzed by SDS-PAGE, rIL-17RC was expressed mainly in the form of inclusion bodies (Figure 1b). Western blotting assays (Figure 1c) showed 55kDa protein band appropriately, further confirmed that rIL-17RC contained His-tag.

The result of binding assay of rIL-17RC protein showed that a colored product was formed in proportion to the amount of rIL-17RC protein in the sample wells; however, none of colored product was observed in the control wells. It was indicated that the prepared rIL-17RC protein could specifically bind to its ligand IL-17A.

(A) PCR amplification of rIL-17RC gene fragments on agarose gel 1.2%. Lane M: 200bp DNA marker; Lane 1–4: recombinant *E.coli* colony by PCR amplification; Lane 5: blank control.

(B) Application of SDS-PAGE in expression detection and purification of rIL-17RC protein in *E. coli* BL21. Lane M: Protein standard marker; Lanes 1 and 3: Total cell lysates of *E. coli* with recombinant pET28a (+)/mIL-17RC plasmid induced by 1 mmol/L IPTG; Lanes 2 and 4: Uninduced *E. coli* with recombinant pET28a (+)/mIL-17RC plasmid; Lane 5: Supernatants of sonicated recombinant *E. coli*; Lane 6: Inclusion body of sonicated recombinant *E. coli*; Lane7: Flow through; Lanes 8 and 9: Wash fractions; Lane10: Elution fractions; Lane 11:Purified rIL-17RC protein

(C) Western-blot analysis of purified rIL-17RC protein using anti-6× His Tag antibody.

### 3.2 Characteristics of nanoparticles

**Figure 2.**
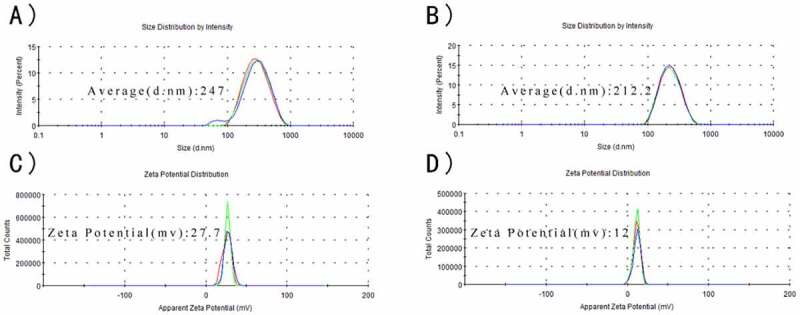
Characteristics of nanoparticles

As observed in Figure 2, CS and CS-RC nanoparticles had the average size of 247 nm and 212.2 nm, respectively, and their zeta potentials were around 27.7 mv and 12 mv, respectively. The polydispersity index (PDI) of CS-RC nanoparticles was 0.148, while CS nanoparticles with PDI of 0.197. The CS-RC nanoparticles presented smooth and spherical morphology, with good dispersibility. The EE of rIL-17RC used to prepare the CS-RC nanoparticles was up to 96.08 ± 3.34%.

Average size of CS nanoparticles (A) and CS-RC nanoparticles (B).

Zeta potentials of nanoparticles (C) and CS-RC nanoparticles (D).

### 3.3 *Biosafety of CS-RC nanoparticles* in vivo

As observed in Table 1, no marked difference was identified among the four groups (n = 6) in the expression of ALT, AST and BUN, as well as in the histopathological changes in their liver, kidney, spleen and lung (data not shown). The body weight of mice in the four groups all gained gradually. The results indicated that CS-RC nanoparticles had a favorable safety profile *in vivo*.

**Table 1.** Expression of ALT, AST, and BUN

Groups	ALT (U/L)	AST (U/L)	BUN (mmol/L)
PBS	5.12 ± 0.87	18.12 ± 1.12	6.01 ± 0.77
rIL-17RC	5.20 ± 0.34	20.45 ± 2.48	6.30 ± 0.30
CS	5.66 ± 1.40	20.66 ± 2.15	5.88 ± 0.53
CS-RC	5.86 ± 0.56	21.54 ± 0.72	6.32 ± 0.48

Note: ALT (0–72.3 U/L), AST (0–72.3 U/L), BUN (0.5–35 U/L)

### 3.4 Effect of CS-RC nanoparticles on cytokines and chemokines expression in BALF

**Figure 3.**
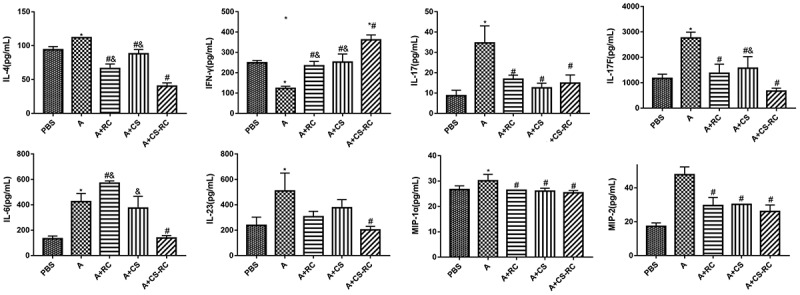
Effect of CS-RC nanoparticles on cytokine and chemokine expression in BALF

To further investigate the effect of CS-RC nanoparticles on cytokine and chemokine expression in the asthma model, ELISA was adopted for measuring the expression of IL-4, IL-17, IL-17F, IL-6, IL-23, MIP-1α, MIP-2, and IFN-γ in BALF (Figure 3). Compared with the A group, the expression of the first seven was significantly decreased after the intervention of CS-RC nanoparticles, while IFN-γ expression was significantly increased. Moreover, significant differences were identified between the A+ RC group and A+ CS-RC group in the expression of IL-4, IL-6, and IFN-γ. Similarly, marked differences were revealed between the A+ CS group and A+ CS-RC group in the expression of IL-4, IL-17F, IFN-γ. However, the levels of IL-17, MIP-1α, MIP-2 showed no significant difference between the three intervention groups, which, respectively, inoculated with CS-RC nanoparticles, CS nanoparticles, rIL-17RC proteins, whereas mice from the three groups demonstrated a significant reduction of IL-17, MIP-1α, MIP-2 in comparison with mice from asthma group (all P < 0.05).

N = 6 per group. *P < 0.05 vs. PBS group, #P < 0.05 vs. A group, &P< 0.05 vs. A+ CS-RC group.

### 3.5 Histopathological analysis of lung tissue

**Figure 4.**
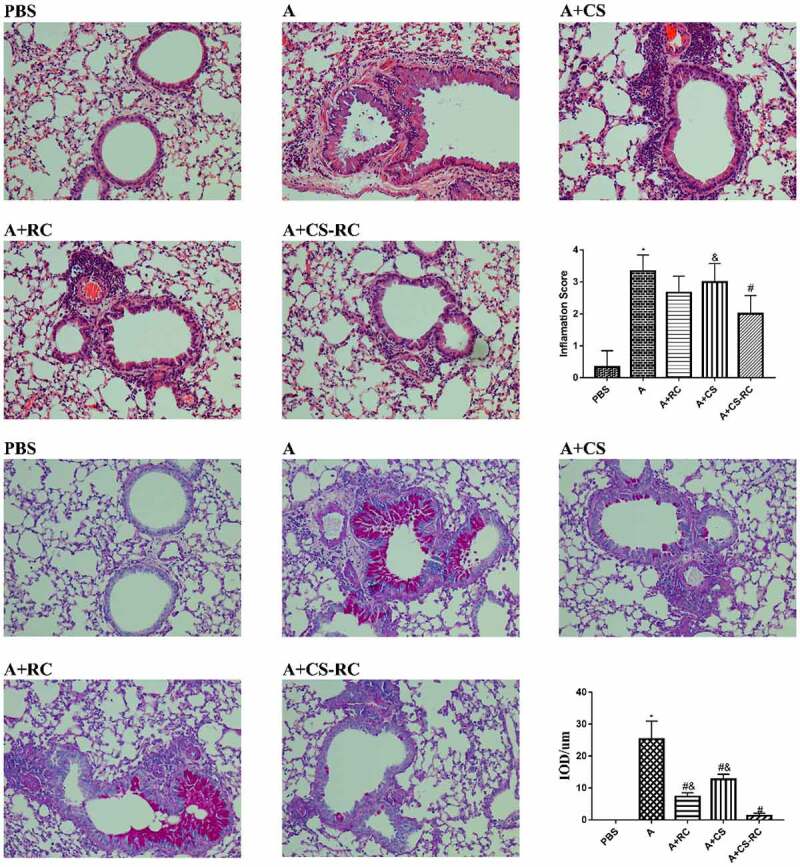
Histopathological analysis of the lung tissues

The effects of CS-RC nanoparticles on airway inﬂammatory and mucus secretion in asthmatic mice were investigated by H&E and PAS staining of lung tissues. In comparison with the PBS group, asthmatic mice showed marked increases in the inflammatory cells infiltrating, airway wall thicken and mucus secretion (Figure 4). These histopathological changes were significantly alleviated after the intervention of CS-RC nanoparticles.

Staining of the lung tissues by H&E and PAS (×200 magnification). As described in the previous report, lung inflammation and goblet cell hyperplasia were analyzed by inflammation score and IOD, respectively, [28]. *P < 0.05 vs. PBS group, #P < 0.05 vs A group, &P < 0.05 vs A+ CS-RC group

## 4. Discussion

Soluble IL-17RC protein, which is a common receptor subunit for IL-17 and IL-17F, has been reported to exist and applied in rheumatoid arthritis via intraperitoneal injection. It can act as a decoy receptor for IL-17 and IL-17F, and inhibit downstream signal transduction, and ultimately prevent various inﬂammatory disorders [31]. Although the crucial roles of IL-17 and IL-17F in allergic airway inflammation have been extensively explored [32], reports on the potential role of their receptor IL-17RC subunit in allergic asthma is limited.

In this study, we generated mouse-soluble IL-17RC protein in a prokaryotic expression system and applied CS nanoparticles as a delivery system to preliminarily evaluated their effect on asthmatic mice by intranasal intervention. To date, research on delivery of soluble IL-17RC protein through CS nanoparticles and their impact on allergic asthma has not been reported.

As expected, the production and purification of rIL-17RC protein were readily available. rIL-17RC protein had a low molecular weight of 55 kDa, which belonged to the FnIII domain of mouse IL-17RC with the removal of its signal peptide. After encapsulation into the CS nanoparticles, the average size and zeta potential of CS-RC nanoparticles were around 212.2 nm and 12 mv, respectively. PDI for nanoparticles typically ranges from 0 to 1; and a PDI value >0.5 leads to aggregation of particles [33,34]. In our study, the PDI of CS-RC nanoparticles was 0.148 leading to good dispersibility *in vivo*. Consistent with previous reports that CS nanoparticles had great protein-binding ability [35,36], the encapsulation efficiency of rIL-17RC proteins was up to 96.08 ± 3.34% in this study. As for the effect of CS-RC nanoparticles *in vivo*, we investigated it via intranasal intervention on asthmatic mice.

In order to explore the optimal intervention dosage of CS-RC nanoparticles, asthmatic mice were intranasally inoculated with different doses of CS-RC nanoparticles. The asthma intervention dosage of 40 μg/mouse (data not shown) was selected and the histopathology of lung tissues observed.

It has been reported that there is a species difference in ligand–receptor interactions between humans and mice. Specifically, in mice, IL-17RC binds preferentially to IL-17F while it binds IL-17F and IL-17A with a similar affinity in human [13,37,38]. Hence, if soluble mouse IL-17RC proteins based on CS nanoparticles could alleviate the inflammatory response of asthmatic mice, it is conceivable that soluble human IL-17RC delivered via a nasal spray in an asthmatic patient might serve as an effective intervention.

The inflammatory processes that underlie allergic asthma are complex and involve an array of cytokines and chemokines [39]. These cytokines include not only Th2, but also Th1 and Th17 cytokines. Among the members of chemokines, macrophage inflammatory protein 1 alpha (MIP-1α) predominantly recruited macrophages and lymphocytes to the site of inflammation. MIP-2 had been shown to exhibit potent neutrophil chemotactic activity [40,41]. In our study, asthma mice showed significantly decreased levels of MIP-1α and MIP-2. The findings suggested that CS-RC nanoparticles intervention might inhibit, at least in part, chemokine expression and inﬂammatory cell infiltration to alleviate inflammatory response in asthma, further confirmed by histopathological changes in lung tissues. In addition, among the three intervention groups, mice in the CS-RC nanoparticles intervention group showed the lowest level of IL-17F, IL-4, IL-6, IL-23, and highest levels of IFN-γ, whereas mice in the CS nanoparticles intervention group showed the lowest level of IL-17. Compared with mice in the rIL-17RC proteins intervention group or CS nanoparticles intervention group, mice treated with CS-RC nanoparticles showed a significant reduction in IL-4 and an increase in IFN-γ. The results indicated the enhancement of immune response to nasally applied rIL-17RC proteins by CS nanoparticles, which was consistent with the immunological adjuvant activity of CS nanoparticles [22]. It had been reported that CS nanoparticles could act as an immunological adjuvant to involve in Th1 and Th2 immune responses [42,43]. In our study, promoting the production of Th1 cytokines IFN-γ was also observed in asthma mice treated with CS nanoparticles. As for the effect on Th2 immune responses of CS nanoparticles, it varied with the differences in experimental conditions [42,43].

As for the underlying delivery mechanisms of CS nanoparticles loaded peptides or proteins, however, it has not been thoroughly understood yet. In combination with previous researches, CS nanoparticles could transiently open the tight junctions between epithelial cells; hence, there is a possibility that CS nanoparticles loaded peptides or proteins might be pass through the nasal epithelium. Furthermore, due to the existence of mucosa-associated lymphoid tissue (MALT) that efficiently induces mucosal and systemic immunity, they might be also triggering the immune reactions from local to distal sites [44,45]. For example, by utilizing the ionic gelation technique, CS nanoparticles (300–400 nm) had been successfully used as insulin carriers via the nose to the systemic circulation [44,46]. Hence, we speculated that CS-RC nanoparticles might reach target sites via MALT and slowly release rIL-17RC proteins, then competitively bind IL-17F and IL-17A with endogenous IL-17 RC, result in the inhibition of downstream signal transduction and thus prevent airway inﬂammation.

## Conclusion

To conclude, airway inflammation in asthma mice could be attenuated after intranasal intervention with soluble rIL-17RC proteins using CS nanoparticles as a delivery system. Therefore, the application of soluble receptor proteins against ligand may be a potentially therapeutic intervention for asthma. However, further studies are necessary to explore the delivery mechanisms underlying CS nanoparticles loaded proteins and the changes of downstream signal transduction after therapeutic intervention.
